# Electromagnetic Navigation in Mandibular Reconstruction Using Autologous Grafts: A Color-Coded Assistance for Precise Positioning of Bone Segments

**DOI:** 10.34133/csbj.0051

**Published:** 2026-04-23

**Authors:** Hagen, Blumenstock, Kühle, Freudlsperger, Kraut, Bouffleur, Knaup, Hoffmann, Eisenmann

**Affiliations:** ^1^ Institute of Medical Informatics, Heidelberg University, Im Neuenheimer Feld 130.3, 69120 Heidelberg, Germany.; ^2^ Department of Oral and Maxillofacial Surgery, Heidelberg University Hospital, Im Neuenheimer Feld 400, 69120 Heidelberg, Germany.

## Abstract

**Background and Objectives:** Virtual surgical planning (VSP) is well established in mandibular reconstruction. However, it is difficult to position bone segments precisely in the operating room due to a lack of visual control and deviating intraoperative conditions. Navigation can help to transfer surgical plans more precisely. We present a color-coded navigation based on electromagnetic tracking that provides simplified 3-dimensional (3D) visualization and real-time feedback to assist with precise bone segment positioning. The aim of this study is to evaluate our approach in model surgeries using 3D-printed bones. **Methods:** We developed software for landmark-based registration and color-coded navigation. Prior to model surgeries, VSP was performed on 11 cases involving hemimandibular defects using in-house software. Osteosynthesis plates were prepared, and bone phantoms and cutting guides were 3D-printed. Mandibular reconstructions were then performed in a laboratory setting using our navigation software. To evaluate applicability and accuracy of our navigation, we compared anatomical and artificial landmarks on the graft via postoperative cone beam computed tomography. **Results:** Navigated mandibular reconstructions were precise compared to VSP, with measured spatial differences of 2.16 ± 1.1 mm at artificial landmarks, 2.95 ± 1.59 mm at the condyle, 2.37 ± 1.4 mm at the jaw angle, and 1.13 ± 0.75 mm at the junction between graft and residual mandible. No substantial differences were found between navigation measurements and cone beam computed tomography. **Conclusions:** Color-coded navigation with simplified 3D visualization of bone segments yielded promising results in model surgery. It has great potential to assist with precise bone segment positioning in mandibular reconstruction.

## Highlights

• Experimental evaluation of in-house developed navigation on 3D-printed bone phantoms
• Electromagnetic navigation for precise mandibular reconstruction
• Color-coded visualization and feedback for simplified bone segment positioning
• High accuracy (2.16 ± 1.1 mm) of mandibular reconstructions in model surgery

## Graphical Abstract

**Figure:**
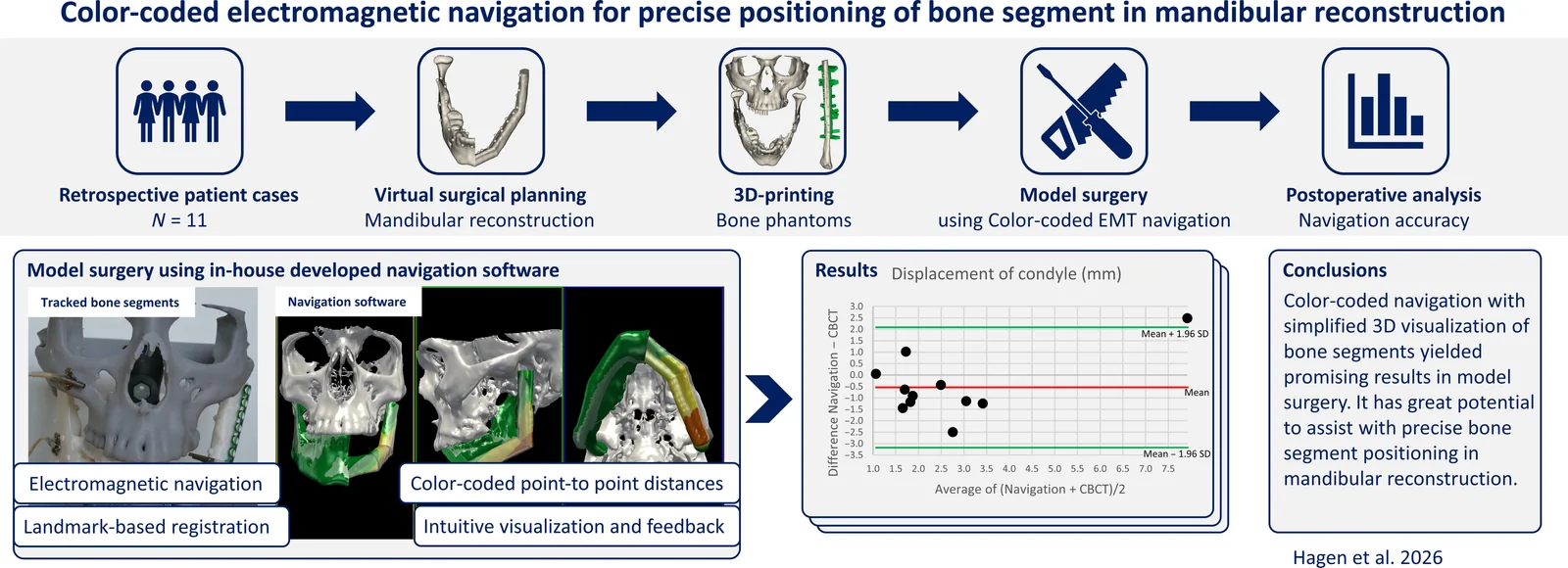

## Introduction

Mandibular reconstruction is primarily indicated for defects after tumor resection but may also be necessary in case of necrosis, inflammation, trauma, or infectious diseases [1]. In recent years, the free fibula flap has become the preferred graft for reconstruction, particularly in long-length defects and cases requiring temporomandibular joint restoration [1–3].

Virtual surgical planning (VSP) is well established in mandibular reconstruction [4–8]. To transfer planning results precisely to the operating room (OR), model surgeries involving manually prebent reconstruction plates, patient-specific implants (PSIs), and cutting guides are applied [4,9–13]. However, despite careful surgical planning, postoperative radiological control sometimes reveals incorrect positioning of the reconstructed condyle or the jaw angle. This can result in functional restrictions like malocclusion, limited mobility, and limited mouth opening, as well as facial asymmetry, graft instability, and chronic pain [2,14–17]. Precise positioning is difficult due to a lack of visual control and intraoperative conditions deviating from the surgical plan—e.g., bone segments do not fit precisely, inaccurate cutting guides resulting in imprecise osteotomies, and rapid tumor growing requiring a more extensive resection [2,16,18–20].

Navigation technology can help surgeons transfer surgical plans more precisely into the OR, thereby increasing accuracy and reducing complications [2,14,19,21,22]. In oral and maxillofacial surgery (OMFS), navigation is used in orthognathic surgery, fracture reconstruction, foreign body removal, skull base surgery, or orbital floor reconstruction [19,23–27]. However, due to the mobility of the mandible, surgical navigation in mandibular reconstruction is more challenging [22].

The literature describes navigation in mandibular reconstruction primarily based on optical tracking. Abbate et al. [28] and Shan et al. [21] report on the position control of cutting guides using a tracked probe. Other approaches use tracked saws and drills to perform precise osteotomies and screw positioning [14,29,30]. Aukema et al. [31] report on a navigated saw slot based on electromagnetic tracking (EMT). In their study, the authors demonstrated angular differences of 2.1 ± 1.4° and length differences of 0.3 ± 0.3 mm compared to the planned segments. Other groups are investigating the use of surgical navigation in robot- and augmented reality (AR)-guided osteotomies [32–37]. To ensure precise positioning of grafts, optical tracked probes are used in plenty of studies to record landmarks on bone segments, which can then be compared to the surgical plan [15,19,21,28,29]. However, these approaches focus on subsequent control of osteotomies and graft. We are not aware of any system that supports graft positioning proactively and provides the surgeon with continuous, real-time feedback. To provide proper navigation assistance, the surgeon needs to quickly grasp the necessary movement of bone segments in 3-dimensional (3D) space. This could reduce uncertainties in mandibular reconstruction and may help to reduce operating times. Additionally, the bulky optical trackers may not be well suited to filigree navigation in OMFS due to limited surgical access. By design, EMT can offer minimalist sensors that may be better suited.

In recent years, we have successfully used EMT for precise bone segment tracking in orthopedics [38,39] and orthognathic surgery [23,40,41]. Building on our previous work, we present EMT-based navigation that provides surgeons with a novel color-coded, real-time feedback to assist with precise graft positioning in mandibular reconstruction. The approach is integrated with our in-house developed VSP software featuring a high degree of automation. It introduces new concepts for registering autologous grafts composed of multiple fibular segments as well as visualization optimized for mandibular reconstruction. The aim of this study is to evaluate this new navigation approach in terms of usability and accuracy in a phantom study using 3D-printed skulls.

## Materials and Methods

The study includes 11 model surgeries to evaluate our EMT navigation approach on additively manufactured skull phantoms of retrospective patient cases of hemimandibular defects including the condyle. All reconstructions are planned using our surgical planning software. Cutting guides and osteosynthesis plates are prepared for each model surgery. The graft, which consists of multiple fibular segments, is positioned and fixed to the residual mandible in the recipient region as a single, integrated unit using our color-coded EMT navigation. Postoperative cone beam computed tomography (CBCT) is performed to examine the surgical accuracy. The study was conducted according to the guidelines of the Declaration of Helsinki and was approved by the local ethics committee of the Medical Faculty of Heidelberg University (S-183/2015). Informed consent was obtained from all subjects involved in the study.

### Virtual surgical planning

For all study cases, tumor resection and mandibular reconstruction are planned utilizing an in-house VSP software that we developed in a previous work [42]. The VSP software is implemented in C++ and based on the Medical Imaging Interaction Toolkit (MITK) (German Cancer Research Center [DKFZ], Heidelberg, Germany). Using a knowledge-based approach, the VSP software enables manual tumor resection planning, automated calculation of reconstruction proposals for autologous fibula grafts—considering relevant functional and aesthetic requirements, and manual adjustment of the calculated reconstruction proposals [42,43].

Based on the VSP results, individual osteosynthesis plates are prepared for each case to ensure the most precise transfer of osteotomies possible. Therefore, our in-house VSP software offers appropriate functionality, including the registration of the osteosynthesis plate onto the virtual surgical plan. Additionally, custom-fit cutting guides with predictive holes (drill guides) are generated for the recipient region and the donor site using self-developed algorithms. Osteotomy margins for saw guide calculation are derived from the VSP results. The registered plate facilitates predicting screw positions and orientations, thereby enabling the precise calculation of drill guides.

### Additive manufacturing of bone phantoms

The skull phantoms for the model surgeries (*N* = 11) are 3D-printed using fused deposition modeling with an Ultimaker S5 (Ultimaker B.V., Utrecht, Netherlands) and an Anycubic i3 Mega S (Anycubic, Shenzhen, China) in a polylactide material. To ensure the initial position of the freely movable mandible (closed bite) during navigation, rigid plug connections between the dental arches of the maxilla and mandible are modeled.

In addition, the fibulae are 3D-printed using fused deposition modeling technology. During model surgery, the planned fibular segments are osteotomized from the manufactured fibulae and assembled into bone grafts. Therefore, the virtually generated cutting guides are 3D-printed from resin (digital light projection) using an Anycubic Photon Mono X (Anycubic, Shenzhen, China). To precisely measure the postoperative accuracy of the mandibular reconstructions, artificial landmarks are added to the surface of each mandible and fibula.

### Tracking technology

**Fig. 1.**
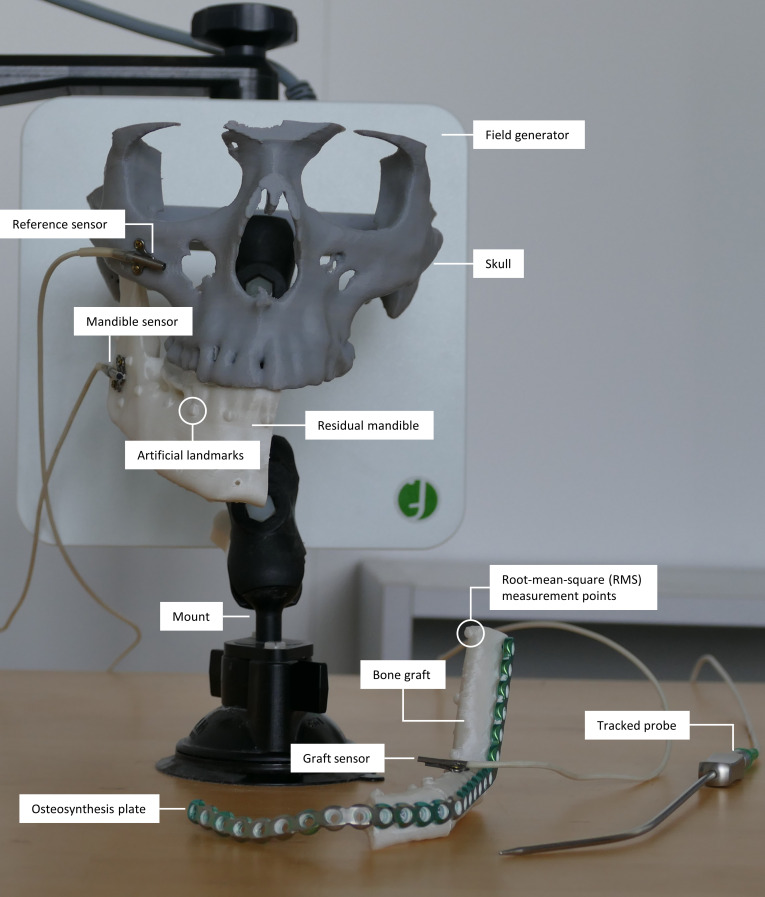
Tracking setup with the tracked pointer probe (bottom right) and fixed electromagnetic sensors on the zygomatic bone (gray), residual mandible (white), and bone graft (white, bottom). The skull phantom is fixed to a custom-made mount. The electromagnetic tracking system’s field generator is positioned behind the skull phantom and close to the surgical site.

Due to limited surgical access and a lack of line of sight, we opted for EMT technology, which we had previously used in a similar setup for orthognathic surgery [23,41]. The measurement volume of about 0.5 × 0.5 × 0.5 m is generated by a field generator of the NDI Aurora system (Northern Digital Inc., Waterloo, Canada) positioned under the patient’s head. To ensure accurate and precise navigation, we use 6-degree-of-freedom (6-DOF) sensors from Fiagon (Fiagon GmbH, Hennigsdorf, Germany). Fiagon offers minimalistic and bone-fixable sensors based on NDI Aurora technology. Compared to established instrument navigation, this enables the navigation of bone segments like the residual mandible and the graft (Fig. 1).

Positioning and orientation of the sensors are measured in relation to the field generator in the tracking system’s coordinate system. For each sensor, real-time measurements of positioning vectors (x-, y-, and z-coordinates) and rotation matrices/quaternions (orientation around x-axes – roll, y-axes – pitch, and z-axes – yaw) are acquired. These data are provided to our navigation software via the tracking system’s interface. This allows for the tracking of bone segment movements across all 6 DOF. By calculating numerous 3D distances relative to the virtual surgical plan, our real-time color coding incorporates all information from the 6-DOF sensors (see the “Bone segment navigation” section).

Mini screws are used to attach 1 sensor to the remaining part of the mandible after tumor resection and 1 sensor to the graft. To allow movement of the patient’s head during surgery, all measurements are taken in reference to a third sensor on the zygomatic bone. All measurements are recorded simultaneously in the coordinate system of the EMT system.

### Implementing the navigation software

Our navigation software is developed in C++ and based on the MITK. MITK provides a broad toolset for computer-assisted surgery as well as the image-guided therapy functionality to support different tracking devices and real-time processing of tracking data [44]. The user interface is implemented via the Qt library.

**Fig. 2.**
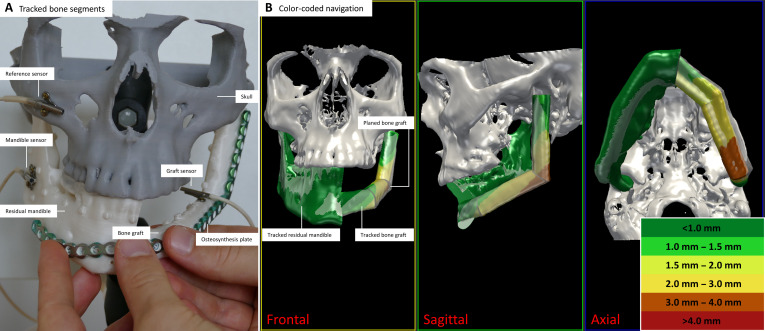
Tracked bone segments and color-coded navigation views. (A) Skull phantom with fixed electromagnetic sensors on the zygomatic bone (gray), residual mandible (white, left), and bone graft (white, right), which is held in place manually (not yet fixed to the residual mandible). (B) Frontal, sagittal, and axial projections of the 3-dimensional (3D) views in the navigation software. The virtual surgical plan is displayed semitransparently. The distances between the tracked bone segments and the planned target position are color-coded, ranging from dark green (<1.0 mm) to dark red (>4.0 mm).

The navigation software provides a dedicated user interface and extends the traditional 4-window view of MITK. It allows for configuration of different visualization views for each phase of navigation to display sliced images (e.g., computed tomography [CT] images) or 3D objects (e.g., bone surfaces). A traditional 4-window view is used for positioning of anatomical landmarks during registration. During navigation, however, 3D visualizations of bone segments from relevant projections (frontal, sagittal, and axial) are displayed (Fig. 2B). Sensor data, time measurements, and registration errors can be saved for postoperative evaluation at any time.

### Registration and measurement of registration errors

The purpose of registration is to superimpose the virtually planned 3D bone segments (residual mandible and graft) onto the corresponding patient anatomy. This enables us to track and visualize the registered 3D bone segments in relation to the respective EMT sensors.

First, suitable anatomical landmarks are positioned in the planning dataset using the navigation software. Corresponding landmarks on the patient are acquired using a tracked pointer probe. The navigation software guides the surgeon through the registration process. To prevent perception problems, both visual and acoustic feedback is provided.

Recording a landmark on the patient begins with holding the pointer still. To prevent minor disturbances, such as a trembling hand, 60 measurement points are acquired at 40 Hz (every 25 ms), and the arithmetic mean is calculated. If a measurement point is recorded too far away (>0.5 mm) from its predecessor, the entire measurement series is discarded and restarted. This ensures that any possible slippage of the pointer is reliably detected.

The actual registration is performed by calculating a transformation matrix using Horn’s method [45]. To evaluate the quality of the registration, the fiducial registration error (FRE) and target registration errors (TREs) are calculated at 2 positions:

• TRE-CONDYLE (at the planned condyle)
• TRE-CENTER (center of gravity of the graft)

If the error values are too high, the surgeon can choose to repeat the registration.

### Bone segment navigation

To ensure smooth navigation, the tracking rate is set to 25 Hz. This allows the tracked 3D bone segments to be compared with planning results and visualized in real time.

The tracked and planned bone segments are represented by the same virtual 3D objects (point clouds with identical shape and size) (Fig. 2). This allows us to determine deviations between bone segments on a very dense point cloud covering the residual mandible and the entire graft. Spatial deviations of both objects are displayed using a color-coding scheme. Therefore, our software calculates point-by-point distances (Euclidean distance) between the point clouds of the tracked and planned object:

$$d\left(p,q\right)=\sqrt{\sum_{i=1}^{3}{\left(q_{i}-p_{i}\right)}^{2}},$$ (1)

*d* = distance

*p* = point in tracked point cloud

*q* = point in planned point cloud

Coloring the point cloud object of the tracked bone segments allows the distances to be displayed for local areas simultaneously. The color scale ranges from dark green (small distances, <1.0 mm) to dark red (large distances, >4.0 mm). Continuous distance calculation and color-coded visualization in different views (frontal, sagittal, and axial) enable us to provide precise navigation assistance (Fig. 2B). The implemented bone segment navigation incorporates all translational and rotational information measured by the 6-DOF sensors. It converts the measured sensor information into a simplified color-coded representation of bone segment displacements (Movie S1). To provide additional orientation, the planned target positions of the graft and residual mandible are displayed semitransparently for the surgeon.

### Model surgery

**Fig. 3.**
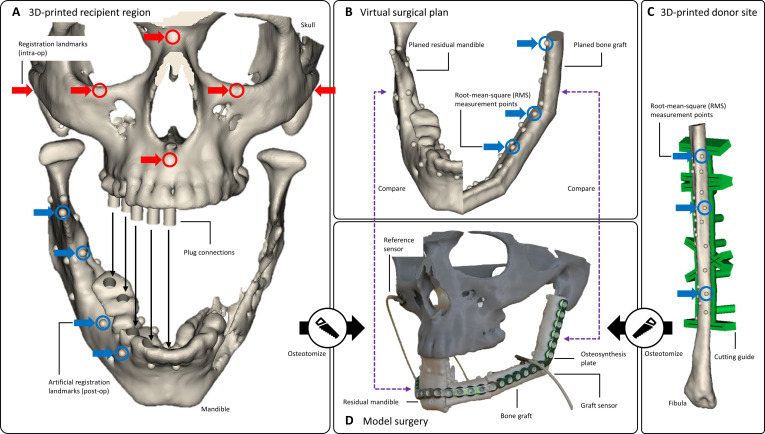
Model surgery procedure. (A) The 3-dimensional (3D)-printed phantoms (skull and mandible) of the recipient region. The anatomical landmarks used for intraoperative (intra-op) registration are displayed in red. The artificial landmarks on the mandible (blue) are used postoperatively (post-op) for registration with cone beam computed tomography (CBCT). The skull and mandible phantoms can be secured using plug connections. (B) The virtual surgical plan for mandibular reconstruction using a 3-segment fibular bone graft. The artificial landmarks on the graft (blue) are used to measure the spatial distances between the planned and implemented graft after surgery. (C) The 3D-printed fibula phantom with corresponding measurement points (blue) derived from the measurement points of the virtually planned bone graft (B). These measurement points on the fibula are required since the graft will be assembled during surgery. The 3D-printed cutting guide for model surgery is displayed in green. (D) The 3D-printed phantoms used for model surgery, with the tracked bone graft fixed to the residual mandible. The residual mandible and the fibular segments of the graft were osteotomized using cutting guides produced from the virtual surgical plan. Throughout the model surgery, the positions of the tracked bone segments (residual mandible and graft) were compared to the virtual surgical plan (B) in real-time (dotted purple lines).

Model surgeries are carried out on a wooden table in a laboratory setting to minimize interferences with the EMT (Fig. 3). However, the instruments and materials used are chosen to simulate surgical conditions as closely as possible. We have conducted similar laboratory and OR studies on EMT navigation accuracy, and we are confident that we can achieve comparable accuracy in the OR [23,38].

At the beginning of each surgical procedure, we secure the 3D-printed skull phantom and mandible in place using the plug connections (Fig. 3A). We attach the reference sensor to the zygomatic bone and the mandibular sensor to the mandible. The mandible is then registered in the same position as in the virtual surgical plan (Fig. 3B). Next, we perform tumor resection using our 3D-printed cutting guides and a water-cooled saw (as used for real interventions) to prevent 3D-printed bone phantoms from thermoplastic deformation.

Using our fibula cutting guide, the planned fibular segments are harvested from the 3D-printed fibula. These fibular segments are then screwed to the plate to form the graft (Fig. 3C and D). The predictive holes in the cutting guides accurately determine position and orientation of the fibular segments in relation to the plate, ensuring high geometric similarity to the virtually planned graft. Once an EMT sensor has been attached, the graft is registered using our landmark-based approach.

With the help of our navigation software, the graft is inserted into the recipient region and fastened to the mandible with surgical screws (Fig. 3B and D). After fixation, we store the final scene of the navigation software, which includes the tracked virtual objects of the graft and mandible, to evaluate navigation accuracy postoperatively.

### Postoperative analysis

**Fig. 4.**
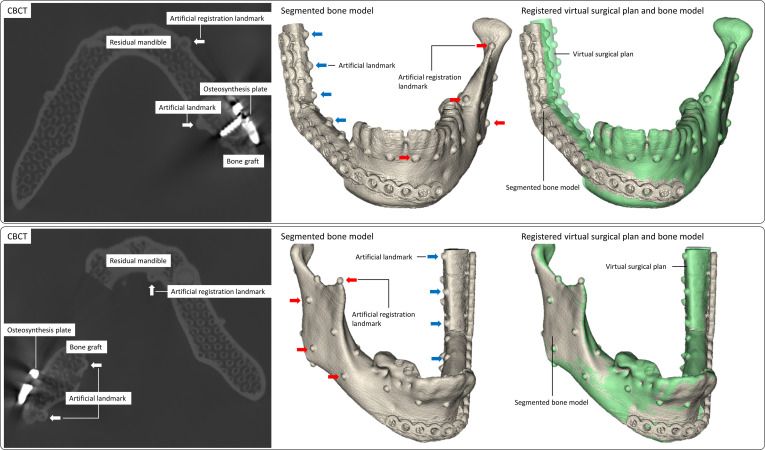
Postoperative imaging. Two study cases reconstructed with 2 fibular segments on the right side of the mandible (top) and the left side of the mandible (bottom). Left: Postoperative cone beam computed tomography (CBCT) scans of the study cases. Middle: Segmented and 3-dimensional (3D) triangulated bone models (gray), with artificial landmarks for measuring accuracy (blue) and artificial registration landmarks (red) of the study cases. Right: Registered virtual surgical plan (green) and bone model (gray) of the study cases.

A CBCT scan of the reconstructed phantom skulls is performed on an Axeos scanner (Dentsply Sirona, Bensheim, Germany) after model surgery to examine navigation accuracy. Cross-sectional images of all study cases are acquired with a pixel spacing of 0.22 mm in plane; 800 rows and columns in plane; a slice thickness of 0.22 mm; and a number of slices of 672. Bone surfaces are segmented using Mimics (Materialise NV, Leuven, Belgium). Registration of segmented bone surfaces from CBCT with the virtual surgical plan is performed using MITK and artificial landmarks on the residual mandible (see the “Additive manufacturing of bone phantoms” section). Figure 4 provides exemplary illustrations of postoperative imaging.

The osteosynthesis plates are made of a highly radiodense material, which causes artifacts in the CBCT. Since the plates are located next to the radiolucent 3D-printed bone models, inaccurate image reconstructions appear on the lateral graft side, as can be seen in the segmentation results (Fig. 4). To reliably determine the accuracy of a model surgery, we use landmarks that are unaffected by artefacts, rather than comparing entire bone segments. Accuracy is measured by the spatial 3D distance between the artificial graft landmarks in the CBCT and the virtual surgical plan. Differences between the measurements of the navigation software and the CBCT at clinically relevant points are analyzed for the condyle (CON), the mandibular angle (ANG), and the junction (JUN) between the graft and the residual mandible. Pearson’s correlation coefficient is used to examine the correlation between navigation and CBCT. Regression analysis and Bland–Altman plots are used to evaluate the degree of agreement between navigation and CBCT.

The usability of the navigation software is evaluated using the System Usability Scale (SUS) [46] and additional statements regarding the registration process and color-coded visualization. The degree of agreement with a statement is rated by the surgeons on a 5-point Likert scale (1 = strongly disagree, 5 = strongly agree).

## Results

All 11 study cases were successfully operated using our navigation software. The average duration of a model surgery was 38.19 ± 9.19 min. Registration of the mandible took 1.21 ± 0.41 min, while registration of the graft took 2.43 ± 1.32 min. For mandible registration, a mean FRE of 1.15 ± 0.25 mm and mean TREs < 1.0 mm were achieved across all cases. The mean FRE during graft registration was 1.94 ± 0.76 mm. The TRE at the condyle was 1.1 ± 0.38 mm. TRE at the graft center was 0.77 ± 0.26 mm, respectively. Individual registration errors are summarized in Table 1.

**Table 1.** Registration errors of the mandible and graft during model surgery for each study case, with the number of attempts (Attempts), the fiducial registration error (FRE), target registration error at the planned condyle (TRE-CONDYLE), and target registration error at the center of gravity of the graft (TRE-CENTER)

Study case	1	2	3	4	5	6	7	8	9	10	11	Mean ± SD
Registration mandible												
Attempts	3	1	1	2	1	1	2	2	1	1	1	1.45 ± 0.69
FRE	1.17	1.42	1.44	1.35	1.14	0.9	0.86	1.14	1.48	1.08	0.72	1.15 ± 0.25
TRE-CONDYLE	0.94	1.14	1.13	1.10	0.84	0.74	0.62	0.84	1.12	0.88	0.59	0.91 ± 0.2
TRE-CENTER	0.88	1.06	1.09	0.95	0.67	0.68	0.49	0.73	0.93	0.86	0.56	0.81 ± 0.2
Registration graft												
Attempts	9	2	1	1	4	1	1	1	8	1	1	2.73 ± 3
FRE	1.51	1.91	1.32	2.01	2.44	1.51	4.0	1.94	1.35	1.64	1.73	1.94 ± 0.76
TRE-CONDYLE	1.25	1.19	0.74	0.99	1.26	0.79	2.12	1.03	0.8	0.97	0.97	1.1 ± 0.38
TRE-CENTER	0.88	0.9	0.57	0.63	0.86	0.52	1.44	0.76	0.55	0.69	0.68	0.77 ± 0.26

The measured spatial 3D difference at the artificial landmarks between postoperative CBCT and virtually planned graft is 2.16 ± 1.1 mm on average. Free-standing fibular segments that reconstruct the ramus mandibulae tend to show greater deviations compared to segments adjacent to the residual mandible (Table 2). This is also evident from the clinically relevant points CON, ANG, and JUN (Table 2): CON exhibits the greatest incorrect positioning (2.95 ± 1.59 mm), compared to ANG (2.37 ± 1.4 mm) and JUN (1.13 ± 0.75 mm).

**Table 2.** Measured spatial differences (in millimeters) between the virtual surgical plan and the cone beam computed tomography (CBCT). Spatial differences at artificial landmarks are indicated for the total graft (Tx) and for each fibular segment (Segments 1 to 4), with Segment 1 being the condyle, and the segment with the highest number being adjacent to the residual mandible. Spatial differences at relevant points are indicated for the condyle (CON), jaw angle (ANG), and the junction between graft and residual mandible (JUN).

Study case	1	2	3	4	5	6	7	8	9	10	11	Mean ± SD
Tx total	1.39	1.82	3.42	4.13	1.96	1.5	3.87	1.35	1.54	1.8	1.02	2.16 ± 1.1
Segment 1	1.79	1.56	3.86	6.08	1.86	2.18	3.82	1.49	1.94	1.93	1.06	
Segment 2	0.81	2.02	3.06	3.86	1.96	1.15	3.98	1.23	1.13	1.65	0.98	
Segment 3				1.43	2.04	0.61	3.72					
Segment 4				1.11								
CON	1.04	2.33	4.01	6.68	2.02	3.62	4.04	2.38	2.71	2.41	1.21	2.95 ± 1.59
ANG	0.93	2.16	4.56	5.43	1.82	1.62	2.91	1.51	1.74	1.69	1.69	2.37 ± 1.4
JUN	1.12	1.91	0.32	0.82	2.45	0.31	2.21	0.76	1.24	0.81	0.5	1.13 ± 0.75

**Fig. 5.**
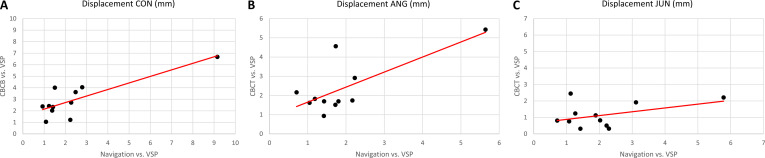
Displacements (in millimeters, mm) and regression line between the navigation measurements compared to virtual surgical planning (VSP) and between the cone beam computed tomography (CBCT) compared to VSP. (A) Displacements at the condyles (CON). (B) Displacements at the jaw angles (ANG). (C) Displacements at the junctions between graft and residual mandible (JUN).

Differences between the measurements of the navigation software and the virtual surgical plan are summarized in Table 3. No substantial differences were found in CON, ANG, or JUN between the navigation displacement measurements (Table 3) and the CBCT (Table 2). Figure 5 illustrates the correlations between navigation and CBCT. The Pearson correlation coefficients of the measured displacements are 0.83 for CON, 0.74 for ANG, and 0.44 for JUN.

**Table 3.** Differences (in millimeters) between the measurements of the navigation software and the virtual surgical plan are indicated for the condyle (CON), jaw angle (ANG), and the junction between graft and residual mandible (JUN)

Study case	1	2	3	4	5	6	7	8	9	10	11	Mean ± SD
CON	1.09	1.42	1.51	9.16	1.38	2.48	2.79	0.93	2.27	1.23	2.23	2.41 ± 2.32
ANG	1.42	0.71	1.73	5.64	1.19	1.05	2.23	1.72	2.17	1.43	1.8	1.92 ± 1.32
JUN	1.89	3.11	2.29	2.02	1.12	1.42	5.78	1.08	1.27	0.72	2.22	2.08 ± 1.41

**Fig. 6.**
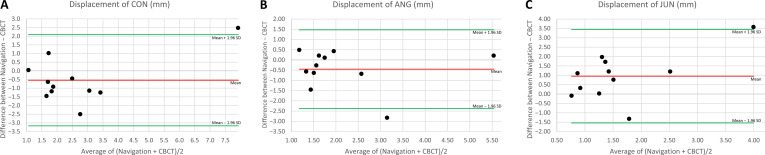
Bland–Altman plots of displacements (in millimeters, mm) between the navigation measurements and cone beam computed tomography (CBCT). The mean is illustrated in red and the limits of agreement in green. (A) Bland–Altman plots for the displacements at the condyles (CON). (B) Bland–Altman plots for the displacements at the jaw angles (ANG). (C) Bland–Altman plots for the displacements at the junctions between graft and residual mandible (JUN).

Differences between the displacement measurements of the navigation software and CBCT are shown in the Bland–Altman plots in Fig. 6. According to the Kolmogorov–Smirnov test, the differences are normally distributed at a 5% significance level, indicating that 95% of the differences fall within the limits of agreement between −3.2 and 2.1 mm for CON, −2.4 and 1.5 mm for ANG, and −1.5 and 3.5 mm for JUN. In 1 study case involving a 4-segment, wide-spanned graft, the measured condyle differences slightly exceeded the upper confidence interval (Fig. 6A). The same case shows maximum displacements of 9.16 mm at CON and 5.64 mm at ANG during navigation, and 6.68 mm at CON and 5.43 mm at ANG in the CBCT (Fig. 5A and B). Maximum difference of 3.57 mm between navigation and CBCT regarding JUN (Fig. 6C) relates to the same case with maximum displacement of 5.78 mm during navigation and 2.21 mm in the CBCT (Fig. 5C).

Two oral and maxillofacial surgeons rated the usability of the navigation software with a SUS score of 82.5 each. SUS scores > 80.3 correspond to grade A or an “excellent” usability. The mandibular registration process was rated at an average of 4.5 (surgeon 1: 4; surgeon 2: 5), while the graft registration process was rated at an average of 4 (surgeon 1: 3; surgeon 2: 5). Surgeon 1 commented that the implemented landmark-based registration of the graft could be challenging at times. Both surgeons rated the suitability of the color-coded visualization for precise graft positioning at 5.

## Discussion

In this work, we present a novel, color-coded navigation approach based on EMT in mandibular reconstruction. Our approach allows for precise bone segment tracking and provides surgeons with intuitive, real-time feedback to assist with graft positioning when surgical access is limited and line of sight is restricted.

Results from the model surgeries show that our landmark-based approach allows for accurate and reliable registration of the patient and virtual surgical plan. However, the graft is pieced together from multiple fibular segments during surgery, which can result in a different shape and adversely affect registration accuracy. Moreover, precise probe positioning on the graft is not always as clear as it is with the anatomical landmarks of the facial skull, because of the fibula’s less distinctive, tubular structure. Accordingly, we observed a learning curve among surgeons during the graft registrations, with a maximum number of 9 attempts for the first case. Due to patient-specific differences, a uniform acceptance level for registration errors has not been specified. However, case 7 stands out with an FRE of 4.0 mm. This major FRE is attributable to shape differences compared to the virtually planed graft. Previous studies in other surgical domains have shown that the fixation of osteosynthesis plates can lead to such undesirable displacements of bone segments [47]. Despite the large FRE, the operating surgeon classified the registration as acceptable due to the reasonable TRE values.

A more efficient and robust graft registration could be achieved through surface scanning. Cuau et al. [48] describe the usage of an optically tracked scanner (structured light) for registering the acquired fibula surface with a preoperative CT scan. Transferring this method to our EMT-based approach is difficult due to the limited measurement volume. Alternatively, the graft surface could be manually scanned with the tracked probe to acquire a large number of surface points for registration. However, an increase in registration time may also increase the ischemia time of the graft.

Postoperative measurements show no substantial differences between electromagnetic (EM) navigation and CBCT. The maximum difference at the junction between graft and residual mandible (JUN) can be explained by a less accurate graft registration (see study case 7). At CON and ANG, slight outliers are evident in a wide-spanning graft composed of 4 fibular segments (see study case 4). We attribute these deviations to the leverage effect of the graft. Minimal shifts or distortions after graft fixation can strongly affect the position of the free-standing condyle. However, the reconstruction of approximately two-thirds of the mandible is rather rare.

A mean deviation of approximately 2 mm was measured between the virtual surgical plan and CBCT at the artificial landmarks. Clinically relevant points CON (approximately 3 mm) and ANG (approximately 2.5 mm) exhibit slightly higher mean deviations. Similar studies report accuracies ranging from 0 to 12.5 mm and from 0.9° to 17.5° [49]. However, the evaluation methods, measurement points, case numbers, and severity levels of the study cases reported are very heterogeneous. Therefore, a meaningful comparison without a standardized evaluation procedure is difficult. Additionally, we used rigid plug connections to fix the freely movable mandible to the maxilla. These adjustments could have led to better results compared to patient studies. However, this simplification seems acceptable at such an early stage of research, given that our main objective is to explore the feasibility and applicability of our new navigation approach.

In our previous work, we focused on imageless EM navigation in orthopedics, where derotation of femoral bone segments were measured, as well as in orthognathic surgery, where jaw repositioning was assisted by displaying quantitative information on translational and rotational displacements [23,39]. In the present work, we demonstrate that image-based, color-coded navigation allows for precise and intuitive graft positioning in model surgeries of varying complexity. Compared to established navigation, which provides spatial distance information and angle measurements, color-coding represents a substantial simplification. It reduces the amount of information required for precise positioning to simple visual feedback. However, our color-coded 3D bone segments are displayed in 2-dimensional projections. Spatial deviations are thus visualized in several views simultaneously, which can be difficult to grasp. Furthermore, constant switching between looking at the surgical site, the instruments, and the navigation view makes eye–hand coordination challenging. All of these difficulties can be categorized as perception problems. Since our software operates exclusively on 3D objects, immersive technologies like AR and mixed reality could be used to provide an enhanced view of the surgical site [50]. Additional support could be given by audio feedback (e.g., increasing the signal frequency when approaching the planned target position).

To the best of our knowledge, we introduced EM navigation with real-time feedback for precise bone segment positioning in mandibular reconstruction for the first time. Many studies describe how to control the position of cutting guides or record anatomical graft landmarks to compare the implemented position with the virtual surgical plan using an optical tracked probe [15,19,21,28,29]. Due to their bulky design and the limited surgical access, we believe that optical trackers are less suitable for mandibular reconstruction than EMT. Brouwer de Koning et al. [18] assessed the accuracy of EM navigation for mandibular reconstructions in 11 patients. They recorded measurements of 2.6 ± 1.5 mm for landmarks on cutting guides and 3.2 ± 1.1 mm for anatomical landmarks. Our results from model surgery demonstrate comparable accuracy, suggesting that EM navigation is suitable to provide precise surgical assistance in mandibular reconstruction.

A few publications describe color-coded feedback in VSP and image-guided surgery. Honigmann et al. [51] report improved treatment through color-coded bone distances during VSP in hand, wrist, and forearm corrective surgery. Heinrich et al. [52] describe how color-coded spatial distances can be used in instrument navigation to reduce potential injury of risk structures. In AR-guided orthognathic surgery, Zelechowski et al. [53] describe a Unity application that uses color-coding to visualize planned insertion positions and drill directions of screws when fixing an osteosynthesis plate. Unlike instrument navigation [52,53], our software tracks moving bone segments. By projecting spatial distances onto the virtual objects of the bone segments, we can create a kind of distance heat map, which allows us to visualize not only single distances but also a multitude of distances across the entire 3D object at various points.

A challenge when using navigation in the OR is the need to adapt from the virtual surgical plan, e.g., if the bone segments do not fit together perfectly. Flexibility could be achieved through navigated saw slots [31] and robot- and AR-guided osteotomies [33,34,36,54], in response to any changes that occur during surgery. Using these methods for validation could pave the way for updating the virtual surgical plan intraoperatively, ensuring accurate navigation even under changed conditions. Moreover, recent studies demonstrated that VSP combined with biomechanical analysis, like finite element analysis (FEA), can be used to asses mechanical stability of the graft and long-term outcomes during surgical planning [55]. Chen et al. [56] reported a framework to create biorealistic FEA models with loads of the surrounding muscles to investigate biomechanics of the mandible and temporomandibular joint. Expanding our in-house VSP software with FEA would be a desirable next development step. In addition to geometric accuracy, which we monitor intraoperatively via our EMT navigation, this could further strengthen and improve surgical success.

Our developed VSP and EM navigation contribute to optimized surgical assistance and personalized medicine. Despite the use of these traditional methods in computer-assisted surgery, efforts are underway to assess the potential of large language models, e.g., as additional decision support in OMFS and for automated image analysis [57,58].

A limitation of our work is the lack of surrounding soft tissue in the 3D-printed bone models. Soft tissue, however, can cover the operative field, making it more difficult for surgeons to access and view the bone structures. Furthermore, the material properties of 3D-printed objects differ from those of natural bone. During model surgery, the rigid fixation of the mandible via plug connections might have improved the accuracy of registration and navigation. To achieve a comparable level of accuracy in patients, a reproducible position of the mandible could be ensured using splints [2,21]. Another limitation is that the model surgeries were performed in an ideal laboratory environment. Although we used surgical instruments for our evaluation to simulate the procedure as realistically as possible, additional disruptive influences like interferences from other devices (e.g., monitors) and distortions caused by metal objects (e.g., operating table) must be considered in the OR. In recent years, the surgical accuracy in mandibular reconstruction has improved using PSIs and cutting guides with predictive holes [4,12]. Since this study focused on navigation, this aspect was not explored further. However, it would be of great interest to conduct a future comparison of PSIs with and without color-coded navigation assistance. The small number of 11 study cases, the fact that gender and age were not considered for this pilot study, and the fact that all cases were operated on by 2 surgeons are further limitations.

In summary, our color-coded EM navigation is based on commercially available tracking hardware intended for medical use. Navigation software is developed using MITK (not a medical device), which offers a wide range of functionalities like a tracking interface, visualization and interaction concepts, etc. We consider this a good foundation for future developments. However, we are currently in an early stage of validation. While our findings indicate the potential for color-coded EM navigation of bone segments on 3D-printed bone models, further research is required to evaluate our approach in patients. On the route to a validated navigation software, the next step is to assess navigation accuracy in the OR by passively measuring the positioning of bone segments. Using passive measurement at this stage can prevent any potential adverse effects on the surgery. However, the efficacy of our color-coded EM navigation must also be demonstrated in a prospective study with active navigation assistance. One possible path to obtaining medical device approval would be to cooperate with an industrial partner in future. In addition, we see great potential in adapting our approach for use in other surgical procedures, particularly in orthognathic and orthopedic surgery.

## Data Availability

The datasets presented in this article are not readily available because of legal restrictions. Requests to access the datasets should be directed to the corresponding author.
